# Mangiferin and Morin Attenuate Oxidative Stress, Mitochondrial Dysfunction, and Neurocytotoxicity, Induced by Amyloid Beta Oligomers

**DOI:** 10.1155/2018/2856063

**Published:** 2018-06-14

**Authors:** Elena Alberdi, María Victoria Sánchez-Gómez, Asier Ruiz, Fabio Cavaliere, Carolina Ortiz-Sanz, Tania Quintela-López, Estibaliz Capetillo-Zarate, Santiago Solé-Domènech, Carlos Matute

**Affiliations:** ^1^Departamento de Neurociencias, Universidad del País Vasco (UPV/EHU), 48940 Leioa, Spain; ^2^Centro de Investigación Biomédica en Red de Enfermedades Neurodegenerativas (CIBERNED), Madrid, Spain; ^3^Achucarro Basque Center for Neuroscience, 48940 Leioa, Spain; ^4^Basque Foundation for Science (Ikerbasque), 48013 Bilbao, Spain; ^5^Department of Biochemistry, Weill Cornell Medical College, 1300 York Avenue, New York, NY 10065, USA

## Abstract

Amyloid beta- (A*β*-) mediated ROS overproduction disrupts intraneuronal redox balance and exacerbates mitochondrial dysfunction which leads to neuronal injury. Polyphenols have been investigated as therapeutic agents that promote neuroprotective effects in experimental models of brain injury and neurodegenerative diseases. The aim of this study was to identify the neuroprotective effects of morin and mangiferin against A*β* oligomers in cultured cortical neurons and organotypic slices as well as their mechanisms of action. Cell death caused by A*β* oligomers in neuronal cultures was decreased in the presence of micromolar concentrations of mangiferin or morin, which in turn attenuated oxidative stress. The neuroprotective effects of antioxidants against A*β* were associated with the reduction of A*β*-induced calcium load to mitochondria; mitochondrial membrane depolarization; and release of cytochrome c from mitochondria, a key trigger of apoptosis. Additionally, we observed that both polyphenols activated the endogenous enzymatic antioxidant system and restored oxidized protein levels. Finally, A*β* induced an impairment of energy homeostasis due to a decreased respiratory capacity that was mitigated by morin and mangiferin. Overall, the beneficial effects of polyphenols in preventing mitochondrial dysfunction and neuronal injury in AD cell models suggest that morin and mangiferin hold promise for the treatment of this neurological disorder.

## 1. Introduction

Alzheimer's disease (AD) is characterized clinically by progressive cognitive decline and neuropathologically by the accumulation of amyloid *β* (A*β*) peptides in extracellular plaques and hyperphosphorylated tau protein in intraneuronal tangles in the brain [1]. In addition to the well-known amyloid fibrils involved in plaque formation, A*β* spontaneously forms small, soluble oligomeric assemblies [2]. These forms are described as mainly responsible for cognitive impairment in the disease [3–5]. A*β* oligomers alter the homeostasis of mitochondrial physiology since they increase mitochondrial calcium levels [6], promote the mitochondrial permeability transition pore opening and release of mitochondrial proapoptotic factors, and consequently cause mitochondrial-dependent neuronal cell death [7].

In addition, oxidative stress is another key feature in the disease [8]. Numerous studies have reported the presence of elevated DNA [9], RNA [10], lipid [11], and protein oxidation [12] in brains of subjects with AD and mild cognitive impairment, suggesting that oxidative stress is an early event in AD. Previous attempts to quench ROS have demonstrated benefits to prevent mitochondrial and neuronal injury in AD patients as well as AD animal and cell models [13–16], suggesting that ROS scavengers hold promise for the treatment of AD. Natural polyphenolic compounds exhibit their antioxidant effect by quenching free radical species and/or promoting endogenous antioxidant capacity. Thus, the antioxidant properties certainly may contribute to their neuroprotective effects. The naturally occurring polyphenols, mangiferin, and morin are known to be powerful antioxidants. Mangiferin is a xantone which is abundantly found in fruits and in the cortex of the stalk of *Mangifera indica* L. (mango) [17], whereas morin is present at relatively high concentrations in the branches of *Morus alba* L. (white mulberry) and red wine [18].

The neuroprotective capacity of these polyfenols has been characterized in *in vitro* and *in vivo* models of ischemic neuronal death involving NMDA receptor overactivation and involves attenuation of receptor-mediated calcium influx and oxidative stress as well as apoptosis [19, 20]. Moreover, in Alzheimer's disease animal models, mangiferin and morin have exhibited neuroprotective properties. In APP/PS1 animal model, mangiferin diminished the inflammatory processes, measured by microglia and astrocyte burdens [21]. In the triple transgenic Alzheimer's disease mouse models, morin was described as a novel inhibitor of GSK3*β* that can reduce tau pathology in vivo [22]. In addition, morin reverses neuropathological and cognitive impairments in APPswe/PS1dE9 mice by targeting multiple pathogenic mechanisms [23].

Here, we explored novel neuroprotective mechanisms of mangiferin and morin in A*β* oligomer-induced neuronal injury. We found that natural polyphenols mitigated the mitochondrial dysfunction by mechanisms that regulated mitochondrial calcium homeostasis, mitochondrial membrane potential, and release to cytosol of proapoptotic cytochrome c. Moreover, morin and mangiferin treatments restored the altered redox homeostasis of antioxidant enzymes in neurons treated with oligomeric A*β*. Consequently, natural polyphenols reduced protein oxidation and reestablished bioenergetic failure in A*β*-treated neurons, contributing to the substantial reduction of neuronal death.

## 2. Materials and Methods

### 2.1. Drugs and Culture Medium

Neurobasal medium, B27 supplement, foetal bovine serum, horse serum, and other culture reagents were from Gibco (Invitrogen, Barcelona, Spain). Receptor antagonists MK801 was obtained from Tocris (Cookson, Bristol, UK). Morin and mangiferin (Mng) were obtained by Sigma (Sigma, St. Louis, MO, USA).

### 2.2. Preparation of Amyloid *β* Peptides

Oligomeric amyloid *β* (A*β*1-42) was prepared as reported previously [24]. Briefly, A*β*1-42 (ABX, Radeberg, Germany) was initially dissolved in hexafluoroisopropanol (HFIP, Sigma, St. Louis, MO, USA) to a concentration of 1 mM. For the aggregation protocol, the peptide was resuspended in dry dimethylsulfoxide (5 mM; Sigma, St. Louis, MO, USA). Hams F-12 (PromoCell, LabClinics, Barcelona, Spain) was added to adjust the final peptide concentration to 100 *μ*M to obtain oligomers (4°C for 24 h). Monomeric A*β* was dissolved in PBS to a concentration of 100 *μ*M.

### 2.3. Cortical Cell Culture and Transfection Procedures

Cortical neurons were obtained from the cortical lobes of E18 Sprague-Dawley rat embryos according to previously described procedures [19]. Neurons were resuspended in B27 Neurobasal medium plus 10% FBS and then seeded onto poly-L-ornithine-coated 48-well plates or glass coverslips (12 mm in diameter) at 1.5 × 10^5^ cells per well. For confocal single-cell imaging experiments, cells were plated onto glass-bottom *μ*-dishes (Ibidi GmbH, Germany). The medium was replaced by serum-free, B27-supplemented Neurobasal medium 24 hours later. The cultures were essentially free of astrocytes and microglia and were maintained at 37°C and 5% CO_2_ as was previously described [25, 26].

For transfection of cells, 4 × 10^6^ rat neurons were transfected in suspension before plating with 3 *μ*g of cDNA using the Rat Neuron Nucleofector kit (Lonza, Switzerland) according to the manufacturer instructions and plated and maintained as described above. Cultures were used at 8–9 days *in vitro*.

### 2.4. Organotypic Slice Culture of Neocortex

Cultures were prepared from coronal cerebral sections (400 *μ*m thick with a McIlwain tissue chopper) of brains from Sprague-Dawley rat pups (5–7 days old) using a modification of the defined method [27]. Two slices were plated on each Millicell CM culture inserts (Millipore Ibérica; Madrid, Spain); maintained in 75% HME 03 (Cell Concept, Berlin, Germany), 2 mM L-glutamine (Sigma; St Louis, MO, USA), 25% horse serum, and 25 mg/ml gentamycin (Sigma; St Louis, MO, USA) for 3 days at 37°C; and then shifted in Neurobasal medium supplemented with 0.5% B27 supplement. Experiments were performed at 7–10 days *in vitro*.

### 2.5. Ca^2+^ Imaging in the Cytosol

For [Ca^2+^]i measurements, neurons were loaded with Fluo-4 AM (1 *μ*M; Molecular Probes, Invitrogen, Barcelona, Spain) in Ca^2+^- and Mg^2+^-free HBSS containing 20 mM HEPES, pH 7.4, 10 mM glucose, 10 *μ*M glycine, and 2 mM CaCl_2_ (incubation buffer) for 30 min at 37°C followed by a 20 min wash to allow de-esterification. For data analysis, a homogeneous population of 15–25 cells was selected in the field of view, and neuronal somata were selected as ROIs. Background values were always subtracted, and data are expressed as *F*/*F*_0_ ± SEM (%) in which *F* represents the fluorescence value for a given time point and *F*_0_ represents the mean of the resting level fluorescence.

### 2.6. Mitochondrial Ca^2+^ Imaging

Neurons transfected with mitochondria-targeted 2mtD4cpv Ca^2+^ indicator [28] were transferred to an incubation buffer (see above) and imaged by a TCS SP8X confocal microscope (Leica, Germany). Cells were excited at 458 nm, and cfp and yfp emissions were acquired for FRET ratio quantification at an acquisition rate of 1 frame/15 s for 5 or 10 min depending on the experiment. For data analysis, a homogeneous population of 5–12 cells was selected in the field of view and neuronal somata were selected as ROIs. Background values were always subtracted, and data are expressed as *R*/*R*_0_ ± SEM (%), in which *R* represents the yfp/cpf fluorescence ratio for a given time point and *R*_0_ represents the mean of the resting FRET ratio.

### 2.7. Cell Viability and Toxicity Assays

Cell toxicity assays were performed as described previously with modifications [6]. Cortical neurons at 8–10 days in culture were exposed for 24 hours to A*β* oligomers. Antagonists and inhibitors were added to the cultures 30 min before A*β* oligomers. Twenty-four hours after drug application, cellular damage was estimated by measuring the level of lactate dehydrogenase released (LDH; Cytotox 96®, Promega, Madison, WI) from damaged cells into the culture media. Data were normalized to the activity of LDH released from vehicle-treated cells (100%) and calculated as a percentage of the control. Results were expressed as the means ± SEM of at least three independent experiments performed in triplicates.

Cortical slice cultures were exposed to A*β* oligomers at 5 *μ*M for 24 h. Antagonists were added to cultures 30 min before the A*β* preparation. Cell death in organotypic cultures was evaluated by using the cellular uptake of propidium iodide (PI). Slices were stained by adding 10 *μ*M PI into the culture for 2 h at 37°C and washed with PBS by two times for 10 min. Slices were fixed with 4% PFA in PBS for 40 min at room temperature. Afterwards, the slices were excited with 510–560 nm light and the emitted fluorescence acquired at 610 nm using a rhodamine filter on an inverted fluorescence microscope (Cell Observer Z1, Zeiss). PI fluorescence images were captured with a Plan NeoFluar 2.5x objective (Zeiss), using an EM CCD camera (Hamamatsu, C9100–13), controlled by the Axio Vision program (Zeiss). Images were analyzed with the ImageJ analysis program (NIH, MD, USA), and PI uptake was expressed as the mean gray value per area total analyzed.

### 2.8. Immunocytochemistry in Cultured Neurons

Cells were preincubated with antioxidants (1 *μ*M, 30 min), treated with A*β* oligomers (5 *μ*M; 2 h), and fixed with 4% paraformaldehyde in PBS 10 min. Immunocytochemistry, using a polyclonal antibody to cytochrome c (Santa Cruz Biotechnology, Santa Cruz, CA), was performed as described previously [29]. Cells were washed twice in PBS for 5 min at RT and permeabilized in PBS containing 0.2% Triton X-100 (TX100) for 30 min, and nonspecific binding sites were blocked in 3% BSA in PBS–0.2% TX100 for 30 min. The primary antibody was diluted 1 : 100 in PBS–0.1% TX100 and 5% NGS and applied overnight at 4°C. Cells were labeled for 2 h at RT with fluorescein-conjugated goat anti-rabbit IgG. In all cases, cells were counterstained with Hoechst 33258 to simultaneously evaluate viable cells. Fluorescence intensity quantification of micrographs was measured using Image J software.

### 2.9. Detection of Protein Carbonyls by Oxyblot

The carbonyl content was determined by the derivatization of protein carbonyl groups with 2,4-dinitrophenylhydrazine (DNPH) leading to the formation of stable dinitrophenyl (DNP) hydrazone adducts, which can be detected by Western blot analysis using anti-DNP antibody. The DNPH derivatization was carried out on 12 *μ*g of neuronal protein extracts for 15 min using Oxyblot Kit (Millipore-Chemicon) following the manufacturer's instruction. The derivatives were then resolved by a broad range SDS-PAGE and transferred to a nitrocellulose membrane (Biorad). After blocking in PBST containing 1% BSA at RT for 60 min, the membranes were washed with PBST and incubated overnight with anti-DNP (1 : 200) at 4°C. The membranes were washed with PBST and incubated with horseradish peroxidase- (HRP-) conjugated anti-rabbit IgG at RT for 45 min. The membranes were washed again with PBST, and the proteins were visualized with the ECL chemiluminescence kit (SuperSignal® West Dura, Thermo Fisher Scientific, Inc, Barcelona, Spain) according to the manufacturer's protocol. Quantification of protein carbonyl content on blots was performed using the automatic band detection and band volume analysis applications of ImageLab 4.1 software.

### 2.10. Measurement of Catalase and Superoxide Dismutase Activity

Neurons were collected by centrifugation, and the cell pellet was sonicated on ice in 70 *μ*l of cold PBS, 1 mM EDTA. Cell lysate was centrifugated at 10,000 ×g for 15 min at 4°C, and the supernatant was assayed for catalase and SOD activity by colorimetric assay procedures following the manufacturer's instructions (OxySelect™ catalase and SOD activity assays kits; Cell Biolabs, Inc, San Diego, CA, USA).

### 2.11. Measurement of Intracellular Reactive Oxygen Species

Neurons were exposed to A*β* oligomers alone or with antioxidants as described. Cells were loaded with CM-H2DCFDA at 30 *μ*M to assay the ROS levels. Calcein-AM (1 *μ*M; Molecular Probes, Invitrogen, Barcelona, Spain) was used to quantify the number of cells within the reading field. Fluorescence was measured using a Synergy-HT fluorimeter (Bio-Tek Instruments Incl, Beverly, MA, USA; excitation at 485 nm, emission at 527 nm). All experiments (*n* ≥ 3) were performed at least in quadruplicate and plotted as means ± SEM.

### 2.12. Analysis of Mitochondrial Membrane Potential

Neurons were exposed to A*β* oligomers alone or in the presence of antioxidants, and changes in mitochondrial membrane potential were monitored by the reduction of JC-1 (Molecular Probes, Invitrogen, Barcelona, Spain), according to the manufacturer protocol. Briefly, after drug treatment, cells were loaded with 3 *μ*M JC-1 for 15 min at 37°C and were washed with HBSS without phenol red two times to eliminate the excess dye. In the cytosol, the monomeric form of this dye fluoresces green (excitation at 485 nm, emission at 527 nm), whereas within the mitochondrial matrix, highly concentrated JC-1 forms aggregates that fluoresce red (excitation at 485 nm, emission at 590 nm). Both JC-1 monomers and aggregates were detectable using a Synergy-HT fluorimeter (Bio-Tek Instruments Incl, Beverly, MA, USA), and the changes in mitochondrial potential were calculated as the red/green ratio in each condition. All experiments (*n* ≥ 3) were performed at least in triplicate and plotted as mean ± SEM.

### 2.13. Measurement of Oxygen Consumption Rate

The oxygen consumption rate (OCR) was analyzed by an XF96 Extracellular Flux Analyzer and XF Cell Mito Stress Test kit (Seahorse Bioscience, Agilent Technologies, Santa Clara, CA, USA) following manufacturer instructions. OCR is measured before and after the addition of inhibitors to derive several parameters of mitochondrial respiration. Initially, the baseline cellular OCR is measured, from which basal respiration can be derived by subtracting nonmitochondrial respiration. Next oligomycin, a complex V inhibitor, is added, and the resulting OCR is used to derive ATP-linked respiration (by subtracting the oligomycin rate from baseline cellular OCR) and proton leak respiration (by subtracting nonmitochondrial respiration from the oligomycin rate). Next carbonyl cyanide-p-trifluoromethoxyphenyl-hydrazon (FCCP), a protonophore, is added to collapse the inner membrane gradient, allowing the ETC to function at its maximal rate, and maximal respiratory capacity is derived by subtracting nonmitochondrial respiration from the FCCP rate. Lastly, antimycin A and rotenone, inhibitors of complexes III and I, are added to shut down ETC function, revealing nonmitochondrial respiration. Neurons (3 × 10^4^ per well) were seeded on a poly-L-ornithine-coated XF96 plate and cultured for 9 days according to protocol as above. One hour before starting experiments, cells were incubated in an XF Base medium (Seahorse Bioscience), containing 1 mM pyruvate, 2 mM glutamine, and 10 mM glucose. Cells were treated with either vehicle, A*β* oligomers (5 *μ*M), morin (1 *μ*M), or mangiferin (1 *μ*M), alone and in combination. For the determination of basal respiration, ATP-linked OCR, and maximal respiration, three baseline recordings were made, followed by the sequential addition of oligomycin (2 *μ*M), FCCP (1 *μ*M), and rotenone/antimycin A (500 nM). Values were normalized to cell viability by calcein measurement per well after XF assay completion.

### 2.14. Data Analysis

All data are expressed as mean ± SEM(*n*), where *n* refers to the number of cultures assayed, each obtained from a different group of animals. In single live-cell imaging experiments, *n* refers to number of cells recorded from at least three independent cultures obtained from different groups of animals. For statistical analysis of the [Ca^2+^]_cyt,_ [Ca^2+^]_mit_, basal line-extracted area under curve was calculated from single-cell imaging time lapse curves. One-way analyses of variance followed by Bonferroni post hoc tests and one-tailed Student's *t*-tests were used unless otherwise indicated. Statistical significance was set at *P* ≤ 0.05.

## 3. Results

### 3.1. Characterization of A*β* Oligomers

**Figure 1:**
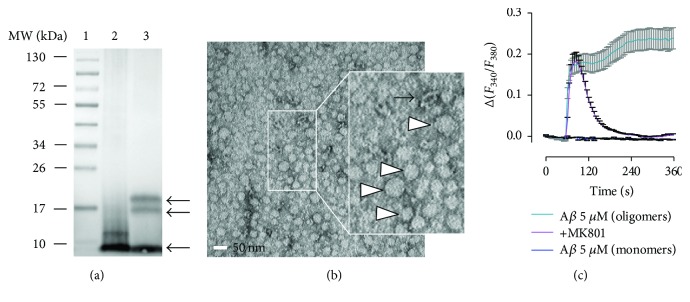
Characterization of oligomeric forms of A*β*1-42 peptide. (a) Representative Western blot showing a broad range of molecular weight protein markers (lane 1), A*β* monomers (lane 2), and A*β* oligomers incubated at 4°C for 24 h (lane 3). A*β* was detected using the monoclonal 6E10 anti-A*β* antibody. Arrows show monomers and oligomers migrating at ~18 kDa. (b) Characterization of A*β* preparation (4°C for 24 h) by TEM showed mainly A*β* oligomers (white arrowheads) and very few protofibrils (black arrow). (c) Ca^2+^ recordings of cultured neurons showed a robust increase in the cytosolic Ca^2+^ concentration induced by A*β* oligomers, but not monomers, both at 5 *μ*M, which is reduced by the NMDA antagonist MK801 (50 *μ*M).

A*β*1-42 peptide rapidly aggregates to form oligomers, protofibrils, and fibrils. To confirm the aggregation state of peptide preparation, a synthetic A*β* sample was subjected to Western blot processing and immunolabeling with 6E10 antibody, which displayed discrete bands corresponding to A*β* monomer, trimer, and tetramer sizes (Figure 1(a)). In addition, transmission electron microscopy (TEM) revealed that synthetic peptide preparation showed a nearly homogenous distribution of round particles which were identified as A*β*1-42 oligomers [30] (Figure 1(b)). Moreover, the differential activity of stable A*β*1-42 oligomers and monomers was found in the dysregulation of neuronal calcium homeostasis. Thus, oligomers, but not monomers, triggered a robust NMDA receptor-dependent calcium response in primary neuronal cultures (Figure 1(c)), as reported earlier [6]. Overall, synthetic A*β* preparation consistently yielded oligomeric forms that selectively activated NMDA receptors expressed in neurons.

### 3.2. Antioxidants Attenuate A*β*-Induced ROS Production and Cell Death in Neurons

**Figure 2:**
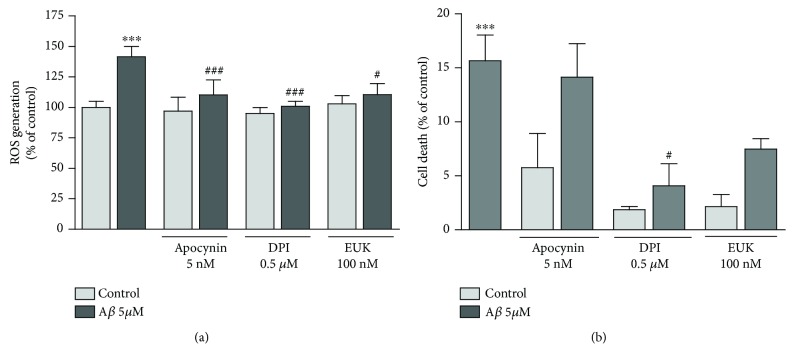
NADPH oxidase inhibitors are neuroprotective against A*β* oligomers. (a) Neurons were treated with *Αβ* (5 *μ*M) for 60 min, and ROS generation was monitored with CM-H2DCFDA (30 *μ*M). Apocynin (5 nM), DPI (0.5 *μ*M), and EUK-134 (100 nM) reduced Α*β*-induced ROS generation in neurons. (b) The toxicity of A*β* oligomers (5 *μ*M) in cultured cortical neurons as measured 24 h later with the LDH viability assay is prevented by the coapplication of A*β* oligomers with NOX inhibitor DPI. Data represent mean ± SEM of the CM-H2DCFDA/calcein and LDH signals in *n* = 4 cultures, expressed as a percentage of control untreated levels (100%). ^∗∗∗^*P* < 0.001 compared with untreated cells; ^###^*P* < 0.001, ^#^*P* < 0.05 compared with A*β*-treated cells.

Excessive production of oxygen-free radicals and other radical species plays an important role in neuronal pathology resulting from A*β* oligomer activity [6, 31]. We examined whether the pharmacological inhibition of ROS could reverse the A*β*-induced neuronal cell death. We found by real-time fluorescence measurements that A*β* oligomers induced ROS generation after 1 hour, which was blocked by compounds that prevent the assembly of NADPH oxidase, apocynin, and diphenyleneiodonium (DPI) (Figure 2(a); 141 ± 8.4%, 110 ± 12%, and 100 ± 4%, respectively; ANOVA, *P* < 0.001; Bonferroni post hoc A*β* versus control, *P* < 0.001; A*β* versus A*β* + apocynin, *P* < 0.001; A*β* versus A*β* + DPI, *P* < 0.001, *n* = 6 cultures). In addition, neurons treated with EUK-134, a mitoprotective antioxidant with superoxide dismutase and catalase activity, showed a robust attenuated oligomeric A*β*-induced signal (102 ± 7%; ANOVA, *P* < 0.001; Bonferroni post hoc A*β* versus A*β* + EUK-134, *P* < 0.05). Treatments of apocynin, DPI, and EUK-134 alone did not modify ROS levels as were compared to vehicle control (Figure 2(a)). Moreover, the DPI rescued cortical neurons from A*β*-induced toxicity (Figure 2(b), 7.5 ± 1% versus 15.7 ± 2.4%, respectively, *n* = 4 cultures; ANOVA, *P* < 0.001; Bonferroni post hoc A*β* versus A*β* + DPI, *P* < 0.05; A*β* versus control, *P* < 0.001). Overall, these results suggest that the reduction of oxidative stress by NADPH oxidase inhibitors is neuroprotective against A*β*-mediated neuronal cell death.

### 3.3. Antioxidant Polyphenols Reduce A*β*-Produced ROS and Neuronal Death in Cultured Neurons

**Figure 3:**
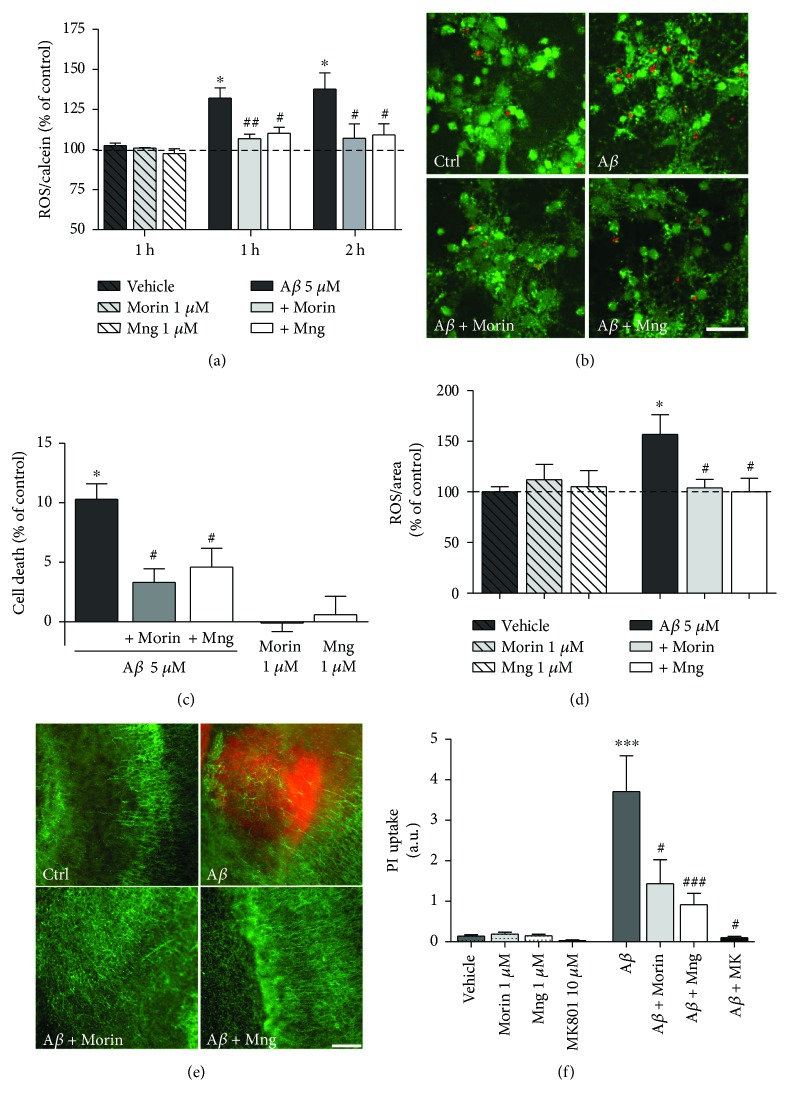
Mangiferin and morin prevent A*β*-induced ROS and neuronal death in dissociated neurons and in cortical organotypic cultures. Cultured neurons (A–C) or cortical organotypic slices (D–F) were incubated with A*β* 5 *μ*M in the presence or absence of polyphenols (1 *μ*M). (a) Mangiferin and morin reduce ROS generation after A*β* stimulus for 1 and 2 h. (b) Photographs show representative fields of calcein and PI fluorescence in cultured neurons displaying cell viability and death, respectively. Scale bar = 50 *μ*M. (c) The toxicity of A*β* oligomers was measured 24 h later with the LDH viability assay. (d) ROS levels in slices, monitored with CM-H2DCFDA after A*β* treatment (30 min) coincubated with morin and mangiferin (1 *μ*M), are shown. (E and F) Representative fields (green MAP-2 and red IP) of cortical organotypic slices and histogram showing A*β* (5 *μ*M, 24 h) toxicity in cultures and protection when oligomers are applied in conjunction with morin (1 *μ*M), mangiferin (1 *μ*M), or MK801 (10 *μ*M). Scale bar in (E) represents 100 *μ*m. Bars represent the mean ± SEM of IP uptake from at least 4–7 cultures per graph, expressed as arbitrary units of mean grey intensity value. ^∗^*P* < 0.05, ^∗∗∗^*P* < 0.001 compared with vehicle-treated cells; ^#^*P* < 0.05, ^##^*P* < 0.01, ^###^*P* < 0.001 compared with A*β*-treated cells.

Mng and morin, two natural antioxidant polyphenols, have a wide spectrum of antioxidant and antiapoptotic properties, which can reduce the mitochondrial dysfunction and neuronal damage associated with the sustained overactivation of glutamate receptor *in vivo* [19] and *in vitro* [20]. Here, we asked whether these two antioxidants might attenuate neuronal oxidative stress and cell death in A*β* oligomer-treated neurons *in vitro* and in cerebral cortex organotypic slices. First, ROS production by A*β* oligomers increased to reach a plateau after 1 hour, which was stayed at the same level for 1 more hour (132 ± 6% and 138 ± 10%, resp., *n* = 4 cultures; ANOVA, *P* < 0.01; Bonferroni post hoc A*β* versus control, *P* < 0.05). ROS levels were attenuated by morin 1 *μ*M and Mng 1 *μ*M (107 ± 3 and 110 ± 4%, resp.; ANOVA, *P* < 0.01; Bonferroni post hoc A*β* versus A*β* + morin, *P* < 0.01; A*β* versus A*β* + Mng *P* < 0.05), showing an effective antioxidant capacity against A*β*-generated oxidative stress (Figure 3(a)). Accordingly, both morin and Mng greatly reduced significantly the A*β* cytotoxicity (5 *μ*M, 24 h) as was revealed by the DNA-binding dye propidium iodide staining (PI; Figure 3(b)) and by lactate dehydrogenase release assays (Figure 3(c); ANOVA, *P* < 0.01; Bonferroni post hoc A*β* versus A*β* + morin and A*β* versus Ab + Mng, *P* < 0.05). Treatments of morin and mangiferin alone did not modify ROS levels and cell viability observed in vehicle control samples (Figure 3(c)).

To further characterize the neuroprotective effects of these antioxidants, we used a more integral preparation, organotypic cultures from the cerebral cortex, to analyze A*β* oligomer-induced ROS and cell death. Slices were prepared to preserve neuronal structural integrity as shown by intense MAP2 staining in the somato-dendritic regions (Figure 3(e)). ROS generation by a short incubation of A*β* for 30 min (157 ± 19, *n* = 4 cultures; ANOVA, *P* < 0.05; Bonferroni post hoc A*β* versus vehicle, *P* < 0.05) were detected in cultured slices. Incubation of slices with morin and Mng reduced the A*β*-induced ROS to control levels (Figure 3(d); 100 ± 10% and 104 ± 2%, *n* = 4 cultures; ANOVA, *P* < 0.05; Bonferroni post hoc A*β* versus A*β* + morin and A*β* versus A*β* + Mng, *P* < 0.05). Moreover, exposure of rat cortical slices to A*β* oligomers for 24 h caused a significant increase of PI uptake by damaged/dead cells, which was strongly reduced by morin and Mng treatment (Figures 3(e) and 3(f); ANOVA *P* < 0.001, Bonferroni post hoc A*β* versus control *P* < 0.001, A*β* versus A*β* + morin, *P* < 0.05; A*β* versus A*β* + Mng, *P* < 0.001, *n* = 4 cultures). The localization of PI staining on Figure 3(e) suggests pyramidal neurons as the most vulnerable cells to A*β* oligomer treatment in cortical slices. In turn, the blockade of NMDA receptors with MK801, used as a positive control, was highly protective as well. Treatments of morin and Mng did not modify the PI uptake observed in vehicle-treatment slices (Figure 3(f)).

These results indicate that the two polyphenol antioxidants protect neurons from A*β* oligomer-induced oxidative stress and toxicity in dissociated cortical neurons as well as in cortical organotypic cultures.

### 3.4. Antioxidant Polyphenols Mitigate A*β*-Induced Mitochondrial Dysfunction in Neurons

**Figure 4:**
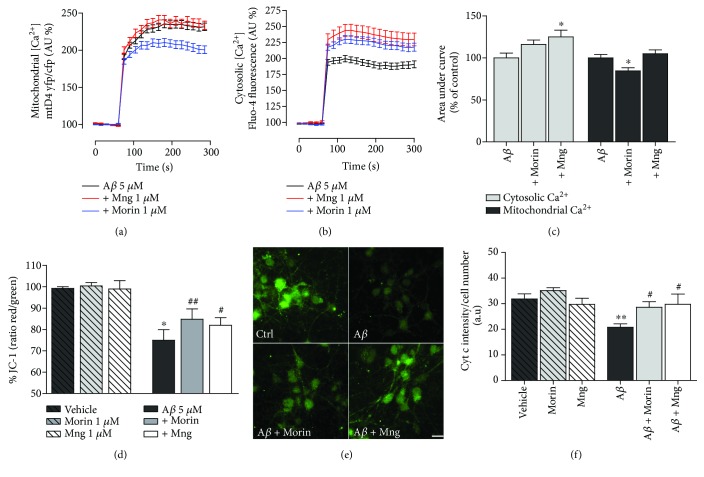
Mangiferin and morin attenuate A*β*-induced mitochondrial dyshomeostasis. (a) A*β* oligomers induce an accumulation of Ca^2+^ in mitochondria of neurons. Cells were transfected with the genetically encoded Ca^2+^ indicator 2mtD4cpv at DIV0, and [Ca^2+^]_mit_ was recorded after 8–10 days in culture. Morin, but not mangiferin, reduces significantly the mitochondrial Ca^2+^ overload. (b) Neurons were loaded with Fluo-4 fluorescence dye and cytosolic [Ca^2+^] changes measured upon the addition of A*β* oligomers. Mangiferin increases the cytosolic calcium levels observed with A*β* oligomers. (A, B) Traces represent the time course of normalized average of fluorescence ± SEM. (c) Graphs illustrate average ± SEM responses of 263 (A) and 187 (B) cells from at least 5 experiments. ^∗^*P* < 0.05, compared to A*β*-treated cells. (d) Morin and mangiferin attenuate mitochondrial membrane depolarization during A*β* stimulation. Cells were treated with A*β* (5 *μ*M, 1 h) after the addition of polyphenols, and the mitochondrial membrane potential was measured using JC-1 fluorescent dye 45 min after A*β* application. Data represent normalized mean ± SEM of the JC-1 red/green fluorescence ratio. ^∗^*P* < 0.05 compared with vehicle-treated cells; ^#^*P* < 0.05, ^##^*P* < 0.01 compared with A*β*-treated cells. (e) Micrographs illustrate cytochrome c immunolabeling in cultured neurons after A*β* treatment (5 *μ*M, 2 h) alone or together with morin and Mng (1 *μ*M). Graph bars represent the intensities of cytochrome c fluorescence normalized to cell number values (average ± SEM, *n* = 3 cultures) displayed as arbitrary units of fluorescence. ^∗∗^*P* < 0.01 compared with vehicle-treated cells; ^#^*P* < 0.05 compared with A*β*-treated cells.

Next, we asked whether these two antioxidants might attenuate mitochondrial damage in A*β* oligomer-treated neurons. First, we observed that neuronal [Ca^2+^]_mit_ increased to 2.35-fold the basal levels following application of A*β* 5 *μ*M. The preincubation of morin 1 *μ*M, but not of mangiferin, reduced significantly the [Ca^2+^]_mit_ overload to twofold (Figures 4(a) and 4(c); ANOVA, *P* < 0.01; Bonferroni post hoc A*β* versus A*β* + morin, *P* > 0.05,). In turn, mangiferin increased the [Ca^2+^]_cyt_ overload induced by A*β* from 2 to 2.3-fold of the basal levels (Figures 4(b) and 4(c); ANOVA, *P* < 0.05; Bonferroni post hoc A*β* versus A*β* + Mng, *P* < 0.05, *n* = 4 cultures). Moreover, morin and mangiferin prevented the loss of mitochondrial membrane potential (Figure 4(d)) and of cytochrome c release to cytosol (Figures 4(e) and 4(f)) induced by A*β* oligomers. The mitochondrial potential was monitored with the fluorescent probe JC-1 and quantified at 1 h of peptide poststimulus. A*β* oligomers induced a reduction in JC-1 fluorescence in neurons (74.9 ± 5%), which was restored by the coincubation of peptides with morin (84.8 ± 5%) and Mng (81.4 ± 3%). Treatments of morin and mangiferin did not modify the mitochondrial potential observed in vehicle-treated cells (Figure 4; ANOVA, *P* < 0.01; Bonferroni post hoc A*β* versus vehicle, *P* < 0.001; A*β* versus A*β* + morin, *P* < 0.01; A*β* versus A*β* + Mng, *P* < 0.05, *n* = 4 cultures).

Similarly, neurons were incubated with A*β* oligomers in the presence or absence of polyphenols as above, and then cytochrome c was visualized by immunocytochemistry. We found that A*β*-treated cells demonstrate a more diffuse and significantly decreased intensity of cytochrome c staining than vehicle-treated cells, suggesting that A*β* oligomers induce cytochrome c release from mitochondria [32]. Treatments with A*β* together morin or Mng restored significantly the cytochrome c fluorescence intensity and the punctuate pattern throughout the cytoplasm suggestive of a mitochondria localization (Figures 4(e) and 4(f); ANOVA, *P* < 0.01; Bonferroni post hoc A*β* versus vehicle, *P* < 0.01; A*β* versus A*β* + morin, *P* < 0.05; A*β* versus A*β* + Mng, *P* < 0.05, *n* = 3 cultures).

Taken together, these results indicate that polyphenol antioxidants reduce [Ca^2+^]_mit_ overload, restore mitochondrial membrane potential, and inhibit cytochrome c release to the cytosol induced by A*β* in neurons.

### 3.5. Morin and Magiferin Restore the A*β*-Reduced Enzymatic Antioxidant Activities and Protein Carbonylation

The antioxidant activity of EUK134 against A*β* oligomer-induced oxidative stress observed in neurons (Figure 2), suggested that a reduced activity of antioxidant enzymes in these cells may underlie A*β*-caused neuronal damage.

**Figure 5:**
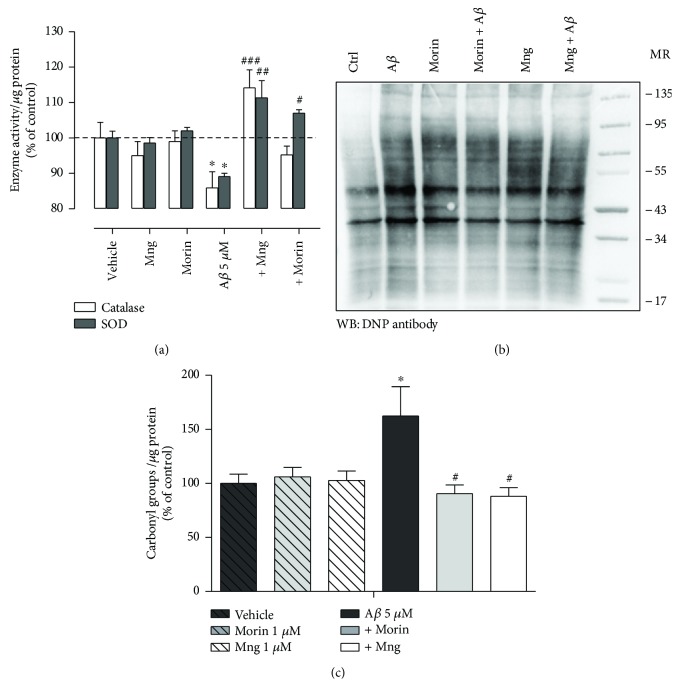
Effects of morin and mangiferin on antioxidant enzyme activities and protein oxidation in A*β*-treated neurons. (a) Neurons were pretreated with morin and mangiferin (1 *μ*M, 30 min) and then exposed to 5 *μ*M A*β* for 2 h. Cells were then harvested and lysed for the determination of levels of catalase and SOD. Data (average ± SEM) from 4 experiments were normalized to the enzyme activity of vehicle-treated cells. ^∗^*P* < 0.05 compared with vehicle-treated cells, ^#^*P* < 0.05, ^##^*P* < 0.01, ^###^*P* < 0.001 compared with A*β*-treated cells. (b, c) Detection of protein carbonyls on neuronal protein extracts was performed by immunoblotting assays based on the use of antidinitrophenyl (DNP) antibody. The Western blot analysis of dinitrophenyl content of 12 *μ*g of proteins illustrates the effects of A*β* (5 *μ*g, 2 h) together with antioxidants in protein carbonyl levels. Graph bars represent the intensities of bands normalized to total protein load (average ± SEM, *n* = 6 cultures) displayed as a percentage of untreated cells. ^∗^*P* < 0.05 compared with untreated cells; ^#^*P* < 0.05 compared with A*β*-treated cells.

Because of that, we verified the effect of A*β* oligomers on the antioxidant capability of neurons. SOD and catalase activities on neurons were significantly weaker after A*β* treatment for 2 h (Figure 5(a); 89 ± 1% and 86 ± 4%, respectively; ANOVA, *P* < 0.001; Bonferroni post hoc A*β* versus vehicle, *P* < 0.05 for both SOD and catalase analysis) than those of vehicle-treated cells. Morin and Mng restored significantly the decreased SOD to 107 ± 2% and 113 ± 5%, respectively (ANOVA, *P* < 0.001; Bonferroni post hoc A*β* versus A*β* + morin, *P* < 0.05; A*β* versus A*β* + Mng, *P* < 0.01), and the catalase activity was significantly restored to 114 ± 5% for Mng (Figure 5(a); ANOVA, *P* < .001; Bonferroni post hoc A*β* versus A*β* + Mng *P* < 0.001).

Among the various oxidative insults to proteins, carbonylation is the most common and severe because of its irreversible and irreparable nature. These derivatives are chemically stable and serve as markers of oxidative stress for most types of ROS. Accordingly, we performed immunoblot analysis using anti-DNP antibody to test protein carbonylation (Figure 5(b), see Methods). Neuronal samples (12 *μ*g) after A*β* treatment (5 *μ*M, 2 h) showed higher levels of total carbonyl content, and morin and Mng antioxidants totally prevented them (153 ± 24%, 89 ± 8%, and 91 ± 8%, respectively; ANOVA, *P* < 0.01; Bonferroni post hoc A*β* versus vehicle, A*β* versus A*β* + morin and A*β* versus A*β* + Mng, *P* < 0.05, *n* = 5 cultures). Morin and Mng alone increased slightly the total carbonyl content of neuronal proteins when levels were compared to them from vehicle-treated cells (100%, Figures 5(b) and 5(c))

Overall, these results showed that morin and Mng may protect neurons against oxidative stress caused by A*β* oligomers by mechanisms which restore the activity of antioxidant enzymes and consequently reduce protein oxidation of neurons.

### 3.6. A*β*-Induced Respiratory Inhibition Is Rescued by Morin and Mangiferin

**Figure 6:**
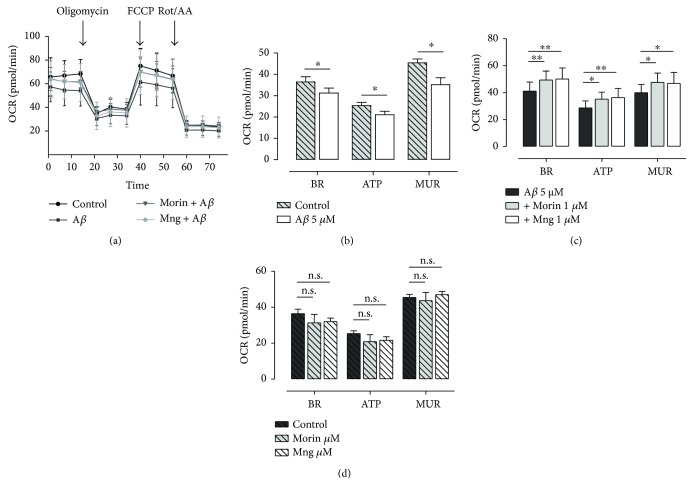
A*β*-induced respiratory inhibition is rescued by morin and mangiferin. (a) Primary neurons in the presence or absence of morin or mangiferin (1 *μ*M) were exposed to vehicle or A*β* (5 *μ*M, 1 h) in XF Base medium (Seahorse Bioscience) containing 1 mM pyruvate, 2 mM glutamine, and 10 mM glucose, and mitochondrial oxygen consumption rate (OCR) was measured using a extracellular flux analyzer (Seahorse XFe96 analyzer). Mitochondrial function in neurons was determined through sequential addition of 2 *μ*M oligomycin, 1 *μ*M FCCP, and rotenone plus antimycin A (both 0.5 *μ*M). This allowed the determination of basal oxygen consumption (BR), oxygen consumption linked to ATP synthesis (ATP) and mitochondrial uncoupled respiration (MUR) (see methods for OCR calculation). (b) A*β* oligomers caused mitochondrial dysfunction. Graph bars represent the average ± SEM of BR, ATP, and MUR OCR in vehicle-treated cells (37 ± 2.5, 25.3 ± 1.5 and 45.4 ± 1.8 pmol/min) versus A*β*-treated cells (31 ± 2, 21 ± 1.5 and 35.2 ± 3 pmol/min), respectively. ^∗^*P* < 0.05, *n* = 5 cultures. (c) Morin and Mng rescued the A*β*-induced mitochondrial respiration inhibition. Graph bars represent the average ± SEM of BR, ATP, and MUR OCR in A*β*-treated, A*β* + morin-treated, and A*β* + Mng-treated cells. ^∗^*P* < 0.05, *P* < 0.01 comparing antioxidant-treated cells with A*β*-treated cells. (d) Morin and Mng treatments did not change significantly the OCR in any parameters of mitochondrial respiration BR, ATP, and MUR (n.s.: not significant).

The regulation of ATP production depends on calcium concentration and respiratory state of mitochondria [33]. Since A*β* oligomers increased the basal levels of [Ca^2+^]_mit_, we next analyzed the impact of A*β* on bioenergetics. O_2_ consumption rate (OCR, see Methods) was measured in vehicle- and A*β*-treated cells before and after the addition of oligomycin to calculate basal respiration (BR) and ATP-linked respiration (ATP) of cells, respectively. Next, FCCP was added to calculate the maximal respiratory capacity (MUR). In all cases, neuronal OCR was inhibited after the addition of A*β* oligomers (Figures 6(a) and 6(b), ^∗^*P* < 0.05 compared to control cells) The coincubation of A*β* oligomers together morin and Mng antioxidants prevented significantly the inhibition of A*β*-induced OCR as is shown in Figure 6(c) (ANOVA, *P* < 0.05; Bonferroni post hoc ^∗^*P* < 0.05, ^∗∗^*P* < 0.01). The treatment of neurons with morin and Mng alone did not significantly change BR, ATP, and MUR (Figure 6(d)).

Overall, the A*β*-induced impairment of energy homeostasis due to a decreased respiratory capacity is blocked by antioxidants morin and mangiferin, a feature which, in turn, may reduce neuron demise.

## 4. Discussion

Under physiological conditions, low levels of ROS are necessary components of signal transduction cascades in a number of functions [34]. However, high levels of ROS, generated when their rate of production exceeds cellular scavenging capacity, are harmful in AD. In addition to oxidative stress, AD is characterized by the disruption of Ca^2+^ homeostasis, mitochondrial dysfunction, and increased sensitivity to apoptosis. All these alterations are involved in A*β* neurotoxicity.

Here, we provided evidence that two antioxidant polyphenols, mangiferin and morin, attenuate oxidative stress, mitochondrial dysfunction, and cell death caused by *Αβ* peptide oligomers in neurons in culture and in cortical organotypic slices. In addition, we showed that both antioxidants restore enzymatic antioxidant activities and consequently mitigate the protein oxidation levels and, importantly, attenuate the impairment of energy homeostasis after A*β* oligomer treatment. These natural polyphenolic compounds could be therefore promising therapeutic tools in AD.

The main cellular sources of ROS are the mitochondria and NADPH oxidases which contribute to rapid ROS generation that in turn is dependent on cytosolic Ca^2+^ load in cortical neurons [35]. Furthermore, crosstalk between the mitochondria and NOX enzymes may represent a feedforward vicious circle of ROS production, which can be pharmacologically targeted under conditions of oxidative stress [36]. In our study, we showed that ROS quenching and neuronal cell death is reduced by NOX enzyme inhibitors, as DPI and apocinin, thus confirming the production of ROS through this enzymatic complex after A*β* stimulation. Previous findings have demonstrated a role for NOX-mediated ROS production in the cytotoxic effects of A*β* on cortical neurons since a specific peptide inhibitor of NOX, Gp91ds-tat, effectively abrogated A*β*-induced ROS production. In this context, a major polyphenolic component of green tea, EGCG [(−)-epigallocatechin-3-gallate], prevented ROS production by NOX and mitochondrial dysfunction demonstrating the therapeutic potential of dietary polyphenols on A*β* toxicity [37]. Furthermore, our results showed that the mitoprotective antioxidant EUK134 robustly reduced ROS levels in neurons treated with A*β* oligomeric, suggesting a causal relationship between mitochondrial ROS imbalance and A*β*-induced impairments. In a similar scenario, the naturally occurring polyphenols morin and mangiferin with oxygen radical scavenging activity [25] reduced significantly the increase in ROS and prevented neuronal demise, which suggests that both flavonoids, in a similar way as EGCG, are potent scavengers of ROS generated by both NOX enzyme activity and mitochondria.

In addition, we speculated that our findings may be related to the capacity of morin and mangiferin to protect the mitochondria from damage that was caused by A*β* oligomers and, in this manner, to break a vicious circle between the ROS sources, described in pathophysiological processes [36]. Indeed, a sustained overactivation of glutamate receptors by A*β* oligomers markedly produces mitochondrial Ca^2+^ overload causing the depolarization of the mitochondrial membrane, ROS generation, and apoptotic neuronal death [6, 38, 39]. The results of the current study show that polyphenols attenuate mitochondrial calcium uptake and depolarization produced by A*β* oligomers. These findings may be related to the capacity of morin to reduce calcium entry via the mitochondria without changes on the Ca^2+^ permeability of glutamate receptor, as was previously showed after excitotoxic insults in cultured neurons with NMDA and AMPA agonists [19, 25]. Previous data have demonstrated that polyphenols may facilitate the balance of cellular Ca^2+^ by modulation Ca^2+^ channel and pump activities on pathophysiological conditions. Specifically, mangiferin prevented methylmercury-mediated Ca^2+^ influx in a human neuroblastoma cell line, showing a neuroprotective potential activity [40]. According to these findings, Roselle polyphenols elicited a negative ionotropic response of agonists for L-type Ca^2+^ channels by possibly modulating calcium entry in cardiac cells [41]. However, in cancer cells, resveratrol and picetannol strongly enhanced the mitochondrial Ca^2+^ uptake by mechanisms involving SERCA activity reduction [42]. Therefore, polyphenol effects on controlling Ca^2+^ mechanisms that are associated with mitochondrial injury may prevent A*β*-induced neuronal demise.

Antiapoptotic activities for antioxidants morin and mangiferin were previously described in neuronal and oligodendroglial excitotoxic cell deaths [19, 25]. Both antioxidants showed the capacity to reduce caspase-3 activation, a cell death effector related to cytosolic cytochrome c. Here, we showed that both antioxidants reduced cytochrome c release from mitochondria membranes to cytosol. The release of cytochrome c from the mitochondria is a key initiative step in the apoptotic process, although the mechanisms regulating the permeabilization of the outer mitochondrial membrane and the release of intermembrane space proteins remain controversial [43]. Cytochrome c is normally bound to the inner mitochondrial membrane by an association with the anionic phospholipid cardiolipin. It seems that the dissociation of cytochrome c from peroxidated cardiolipin might be a critical first step for cytochrome c release into the cytosol and activation of the caspase cascade [44, 45]. A plausible explanation for the antiapoptotic effects reported here for morin and mangiferin is the ability to maintain the homeostasis of the enzymatic antioxidant system after A*β*-oligomeric neuronal injury (Figure 5(a)), a feature that was also described in excitotoxic events [20]. Additionally, mangiferin is capable of chelating iron, avoiding its participation in the Fenton reaction, and preventing lipid peroxidation induced by iron more efficiently than that induced by peroxide [46]. In contrast, morin can inhibit xanthine oxidase, reducing the production of ROS [18] and radicals derived from nitrogen [47]. Thus, antioxidant properties and restoration of enzymatic antioxidant activities by morin and mangiferin might explain their antiapoptotic effects.

Among a wide range of ROS-derived modifications, biomolecule carbonylation is known to be a major hallmark of oxidative stress [48]. Carbonyl stress, characterized by the accumulation of reactive carbonylated species and their reactivity toward nucleophilic substrates, results in biomolecule malfunctions and increased toxicity and can finally lead to apoptotic cell death [49]. Further evidence to support the role of protein carbonylation in the pathogenesis of human disorders has provided a strong link between disease onset/progression and oxidative stress. In our study, we found that the acute treatment of neurons with A*β* oligomers increased levels of protein carbonyls of cultured neurons and that morin and mangiferin prevented them. Furthermore, protein carbonyls were observed in the hippocampi of the triple transgenic Alzheimer's disease mice as compared to the nontransgenic controls [50]. In addition, the majority of carbonylated proteins identified by redox proteomics were found in CSF at early stages of AD. Thus, oxidatively modified CSF proteins are already present in mild cognitive impairment compared with controls and remain oxidized in late AD, thus suggesting that the dysfunction of selected proteins initiate many years before severe dementia occurs [51]. Therefore, antioxidants such morin and mangiferin, which reduced carbonyl stress in the Alzheimer's disease, hold promise for early treatment of the disease.

Another key finding of this study was that A*β* oligomers promote a functional energetic decline affecting the mitochondrial basal respiration; oligomycin-sensitive respiration or ATP turnover; and the maximal respiration in the presence of FCCP, resulting in neuronal energy deficits. In this scenario, morin and mangiferin restored the cell respiratory control, the predominant physiological function of mitochondria. According to these findings, polyphenolic compounds EGCG and resveratrol reversed severe impairments of mitochondrial bioenergetics of hippocampal progenitor cells in Ts65Dn mice, a severe trisomic Down Syndrome mouse model, promoting neuronal progenitors cell proliferation [52]. Overall, the neuroprotective role of morin and mangiferin may derive essentially from its ability to reactivate mitochondrial bioenergetics.

## 5. Conclusion

In summary, we described in this study that morin and mangiferin strongly protect against A*β*-induced mitochondrial dysfunction and neuronal cell death. Specifically, we provided evidence showing clearly that these two natural antioxidants preserve cell respiration, promote detoxification of reactive oxygen species, protect from some forms of apoptosis, and regulate mitochondrial matrix calcium in neurons exposed to A*β*. Together, these results strongly suggest that morin and mangiferin are promising therapeutic tools to restore mitochondrial functions and redox homeostasis in AD.
